# Primary Cutaneous Myxoid Spindle Cell Squamous Cell Carcinoma of the Scalp: A Case Report

**DOI:** 10.5146/tjpath.2020.01499

**Published:** 2021-01-15

**Authors:** Recep Bedir, Mehpare Suntur, Orhan Semerci

**Affiliations:** Department of Pathology, Recep Tayyip Erdoğan University, School of Medicine, Rize, Turkey

**Keywords:** Squamous cell carcinomas, Cutaneous, Myxoid spindle cell variant

## Abstract

Cutaneous squamous cell carcinomas are the second most frequent type of non-melanoma skin cancer. A 78-year-old man with a slow-growing but large nodular lesion on his scalp presented to the hospital. The nodular lesion was excised. Histologically, the lesion was diagnosed as a primary cutaneous myxoid spindle cell squamous cell carcinoma, which is the subject of this case report. Extracellular mucin production is an even less common finding in SCC. We also aim to discuss the histological and immunohistochemical findings for distinguishing MSC SCC from other primary cutaneous and metastatic spindle cell neoplasms with myxoid stroma.

## INTRODUCTION

Primary cutaneous myxoid spindle cell squamous cell carcinoma (MSC SCC) was first identified by Yang et al. (1). This rare variant of SCC has been reported in only 7 cases in the literature to date (2). Here, we present an MSC SCC case located on the scalp of a 78-year-old male patient by employing immunohistochemical analysis for the differential diagnosis.

## CASE REPORT

**Figure 1:**
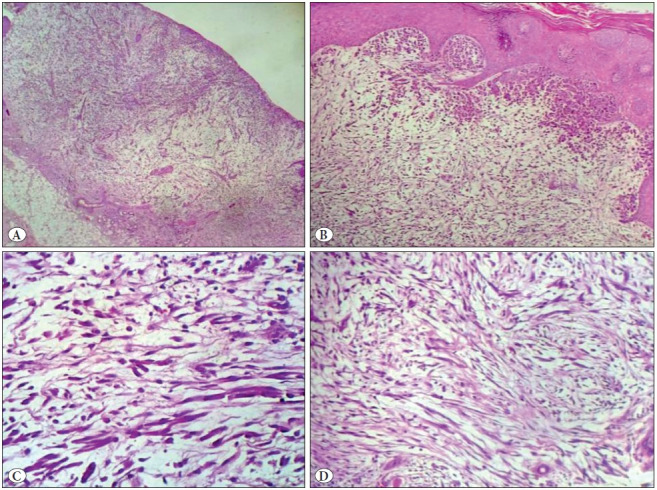
**A)** Tumor showing infiltration as single cells and cell groups in prominent myxomatous stroma of the dermis and ulceration in the epidermis (H&E; x200). **B)** The tumor consists of atypical epithelial cells with spindle-like morphology and plasmacytoid appearance (H&E; x400). **C)** Tumor showing spindle cell proliferation with prominent myxoid stroma (H&E; x400). **D)** Tumor showing storiform pattern mimicking a myxoid sarcoma (H&E; x400).

**Figure 2:**
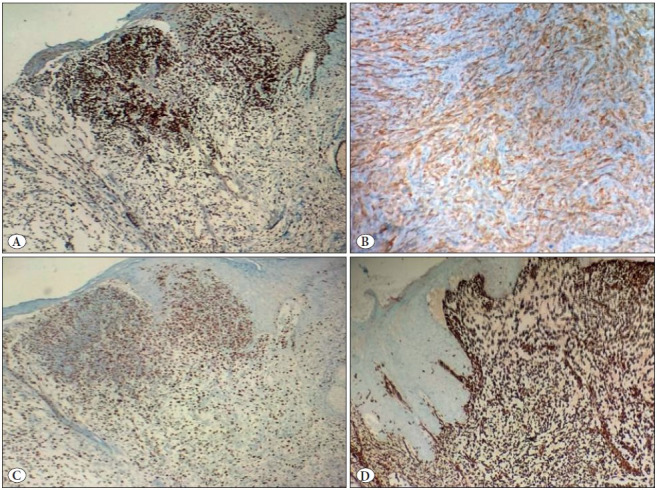
**A)** The tumor showed diffuse positivity for p40 (IHC; x200). **B)** The tumor showed diffuse positivity for AE1/3 (IHC; x200). **C)** The tumor showed a high Ki-67 proliferation index (IHC; x200). **D)** The tumor was diffuse positive for vimentin (IHC; x400).

**Figure 3:**
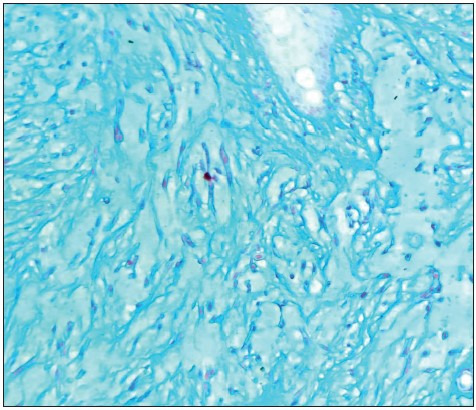
Alcian blue staining was positive in the myxoid stroma (Alcian blue; x200).

A 78-year-old male presented to the clinic with an ulcerated nodular lesion at the parietal region of the scalp that had failed to heal and had become gradually larger in the last 2 years. The lesion was removed by wide surgical excision. An ulcerated nodular lesion with a diameter of 0.5 cm was macroscopically observed on the cross-sectional surface of the 0.6 cm-thick tissue covered with skin 1 cm in diameter. Microscopic examination revealed a tumor with infiltration of single cells/multiple groups of cells and cell groups in prominent myxomatous stroma of the dermis and ulceration in the epidermis. The tumor comprised atypical epithelial cells with a spindle-like plasmacytoid appearance and large hyperchromatic nuclei with prominent nucleoli. The storiform pattern of the tumor mimicked myxoid sarcoma (Figure 1). There was mild pleomorphism in the neoplastic cells, increased mitotic activity, and peri-neural invasion, but no necrosis or lymphovascular invasion. No tumor was observed within the surgical limits. The invasion depth of the tumor was 0.3 cm with a diameter of 0.7 cm (stage T1). Immunohistochemical examination for the differential diagnosis of malignant melanoma and mesenchymal tumors revealed negative staining in the neoplastic cells for S-100, HMB-45, MART1, SMA, and desmin. The neoplastic cells showed diffuse staining with Pan-cytokeratin (AE1/3), P40, and vimentin (Figure 2). The Ki-67 proliferation index of the tumor was high (40-50%) (Figure 2). Alcian blue staining was positive in the myxoid stroma (Figure 3). No intracellular mucin accumulation was observed with the Periodic Acid-Schiff stain in combination with diastase (PAS-D). Based on these results, the case was diagnosed as MSC SCC. No enlarged pathological lymph node was observed on ultrasonography (USG) examination of the head and neck region. No additional treatment was needed for the patient and follow-up was recommended.

Written informed consent was obtained from the patient who was presented in this case report.

## DISCUSSION

Intracellular mucin production in cutaneous MSC SCC is rarely seen and this condition is named “adenosquamous carcinoma” that is a more aggressive variant of SCC. Stromal mucin in SCC is rare (1–6). Primary cutaneous SCC is the second most frequently encountered skin cancer and has many subtypes. Cutaneous spindle cell SCC is a rarely seen variant and has lower risk of metastasis. This risk increases in relation to previous skin damage and immune suppression (3). The sarcomatoid carcinomas on the mucosal surface have a worse prognosis than those with cutaneous localization (7).

Yang et al. (1) have identified the histological criteria of the diagnosis of MSC SCC by using 6 cases. The inclusion criteria of MSC SCC include significant myxoid stromal changes occurring in more than half of the lesion, positive staining for neoplastic spindle (and squamous) cells with a minimum of one cytokeratin (CK) and especially high molecular weight CK (HMWCK), and negative staining for melanocytic and mesenchymal markers of neoplastic cells (S-100, MART-1, actin, myogenin, and desmin). The first 3 criteria were met in all 6 cases. Similar to the histological criteria identified by Yang et al., our case showed negative staining with mesencyhmal and melanocytic markers and positive staining with epithelial markers, and there was mucin production in more than half of the lesion. Since MSS SCC is a rarely seen type of carcinoma, it is very difficult to determine the prognosis. Local recurrence was identified in a limited number of cases by Yang et al. and metastasis was observed in one case. They reported that the invasion depth of the tumor was the most important indicator of an aggressive course (1).

The differential diagnosis of MSS SCC includes myxoid sarcomas (such as myxofibrosarcoma and malignant peripheral nerve sheath tumor), spindle cell atypical fibroxanthoma (AFX) and spindle cell melanoma. The negativity of melanocytic and mesenchymal markers excludes these tumors. However, only vimentin among the mesenchymal markers shows positive staining in spindle cell SCC (1–3).

MSC SCC differs from other cutaneous and mucocutaneous SCCs. MSC SCC lacks mucin-containing glandular structures in contrast to adenosquamous SCC. Adenosquamous carcinoma SCC is characterized by the presence of squamous cells, mucin-producing cells and, in some cases, glandular structures (3,7,8). The intracellular mucin in adenosquamous carcinoma is epithelial mucin (sialomucin) and stains with mucicarmine, and the glandular structures express CEA when present (3). MSC SCC is characterized by a mucinous (myxoid) stroma that is positive with Alcian blue but true epithelial type mucin is not observed (mucicarmine should be negative). In addition, signet ring-like cells were a minor component but no intracytoplasmic mucin was detected within these tumor cells. Thus, unlike cutaneous SCC with mucinous metaplasia (also referred to as signet ring cell SCC), MSC SCC lacks signet ring cells with intracytoplasmic mucin (8).

The chronic long-term exposure to ultraviolet (UV) radiation has been reported to be responsible for the pathogenesis of most SCCs (5). It has been reported that long-term UV exposure likely plays the key instigator role in the epithelial-mesencyhmal transition pathways causing the phenotypic changes observed in spindle cell SCC. HPV plays a major role in SCCs localized in the genital region. Although HPV DNA can be found in 45% of all penile and vulvar SCCs, the highest incidence of HPV DNA is seen in basaloid-type penile SCC cases (9). In our case, the condition was localized to the scalp and there was exposure to UV radiation.

In conclusion, we describe an unusual variant of spindle cell SCC with prominent myxoid features that may mimic myxoid AFX, myxoid sarcomas and, less frequently, melanoma with myxoid change. The differential diagnosis of spindle cell SCCs with myxoid stroma should be made by using melanocytic and mesenchymal markers in order to distinguish them from all the other potential tumors, and the diagnosis should be confirmed by using epithelial markers. Moreover, the patients should be followed up for a long time in terms of recurrence and metastasis.

## CONFLICT of INTEREST

There are no conflicts of interest.
